# Human HLTF mediates postreplication repair by its HIRAN domain-dependent replication fork remodelling

**DOI:** 10.1093/nar/gkv896

**Published:** 2015-09-08

**Authors:** Yathish Jagadheesh Achar, David Balogh, Dante Neculai, Szilvia Juhasz, Monika Morocz, Himabindu Gali, Sirano Dhe-Paganon, Česlovas Venclovas, Lajos Haracska

**Affiliations:** 1 Institute of Genetics, Biological Research Centre, Hungarian Academy of Sciences, Szeged, Temesvari krt. 62, H-6726, Hungary; 2 Zhejiang University, Yuhangtang Road 866, Hangzhou 310058, China; 3 Department of Cancer Biology, Dana Farber Cancer Institute, 450 Brookline Avenue - LC-3310, Boston, MA 02215, USA; 4 Institute of Biotechnology, Vilnius University, Graičiūno 8, Vilnius LT-02241, Lithuania

## Abstract

Defects in the ability to respond properly to an unrepaired DNA lesion blocking replication promote genomic instability and cancer. Human HLTF, implicated in error-free replication of damaged DNA and tumour suppression, exhibits a HIRAN domain, a RING domain, and a SWI/SNF domain facilitating DNA-binding, PCNA-polyubiquitin-ligase, and dsDNA-translocase activities, respectively. Here, we investigate the mechanism of HLTF action with emphasis on its HIRAN domain. We found that in cells HLTF promotes the filling-in of gaps left opposite damaged DNA during replication, and this postreplication repair function depends on its HIRAN domain. Our biochemical assays show that HIRAN domain mutant HLTF proteins retain their ubiquitin ligase, ATPase and dsDNA translocase activities but are impaired in binding to a model replication fork. These data and our structural study indicate that the HIRAN domain recruits HLTF to a stalled replication fork, and it also provides the direction for the movement of the dsDNA translocase motor domain for fork reversal. In more general terms, we suggest functional similarities between the HIRAN, the OB, the HARP2, and other domains found in certain motor proteins, which may explain why only a subset of DNA translocases can carry out fork reversal.

## INTRODUCTION

Unrepaired DNA lesions can block the movement of the replication machinery leading to DNA strand breaks and chromosomal rearrangements providing eventually the driving force to cancer (1). To minimize these consequences, cells possess distinct mechanisms for replicating through damaged DNA such as translesion synthesis or template switching (2–4). During translesion synthesis, one of the low-fidelity translesion synthesis (TLS) polymerases takes over the primer 3′-end from the stalled high-fidelity replicative polymerase and incorporates either a correct or an incorrect nucleotide opposite the damaged base (5). In order to do so, TLS polymerases exhibit flexible active sites, which enable them to cope with various damaged bases on the template (6). However, not all types of lesions in the template strand can be accommodated into the active site of TLS polymerases, and thus they cannot provide a general solution for replication through all types of DNA damage (2,7). The other drawback of TLS polymerases is that they frequently generate mutations. By contrast, template switching provides a more universal solution for the bypass of a wide variety of lesions with the undoubted advantage of operating in an inherently error-free mode. A prerequisite of template switching is the uncoupling of the leading and lagging strand synthesis resulting in the leading strand stalled at the lesion and the lagging strand synthesis going on. Next, remodelling of the stalled nascent leading strand from the damaged template to the newly synthesized strand of the undamaged sister duplex results in a new primer/template structure where a canonical DNA polymerase can extend the primer without encountering the damage (8–10). Depending on the intermediate DNA structure, two alternative mechanisms have been proposed for template switching. The first includes a D-loop intermediate similar to the one proposed for recombination and the other is the so called ‘chicken foot’ model termed after the shape of the intermediate DNA structure. To generate the four-stranded chicken foot, both the leading and the lagging nascent strands at the replication fork should be displaced from the original leading and lagging strand templates and paired together by the reversal of the replication fork (11).

The discovery of the fork reversal activity of yeast Rad5 and the conclusion that this activity is for mediating error-free lesion bypass was in keeping with genetic observations made with various *rad5* mutations (12–15). First, epistasis analysis revealed that RAD5 belongs to the so-called RAD6-RAD18 epistasis group of the DNA damage tolerance pathway, in which it constitutes an error-free branch in addition to the two other TLS polymerase-dependent branches. Deleting *RAD5* inactivates the majority of template switching-dependent damage bypass in yeast cells indicating that Rad5 is a main player in fork rescue via template switching (3,12). Besides Rad5, Mph1 is also shown to mediate replication fork reversal (16,17). Human cells contain two RAD5 homologues, HLTF and SHPRH, which supress mutagenesis in a damage-specific manner (18–21). Furthermore, HLTF and SHPRH are frequently inactivated in various cancers, and their loss increases the frequency of chromosome abnormalities during DNA damage indicating their importance in the maintenance of genomic stability and cancer suppression (22). Of the two human homologues, only HLTF exhibits all the main motifs and enzymatic activities of Rad5. First, depending on its RING domain, HLTF acts as a ubiquitin ligase stimulating Mms2-Ubc13-dependent Lys-63-linked polyubiquitylation of PCNA after its monoubiquitination by Rad6–Rad18 at its K-164 residue (20,23). Furthermore, HLTF exhibits a SWI/SNF helicase domain enabling a dsDNA-dependent ATPase activity for translocation on dsDNA (24). In the course of translocation on DNA, HLTF can displace DNA-bound proteins providing a protein-clearing function at the stalled replication fork (25). Also, HLTF can promote strand invasion and formation of a D-loop structure (26). Finally, depending on the SWI/SNF helicase domain, HLTF has a replication fork reversal activity to generate chicken foot for template switching (24). While the biochemical features and the *in vivo* significance of the RING and the SWI/SNF domains of HLTF have been characterized, much less is known about its HIRAN domain. The HIRAN domain can also be found in bacteria, as a standalone protein or fused to other catalytic domains such as phosphoesterases or nucleases and is suggested to be a DNA-binding domain able to recognize 3′-end of ssDNA (27–29). Nevertheless, to what extent the HIRAN domain contributes to the DNA damage tolerance function has remained unknown.

Although RAD5 is known to be a dominant player in yeast postreplication repair (PRR) (3,12), the role of HLTF and the degree of its contribution to human PRR has not been clarified. Postreplication repair ensures the full completion of the replication by facilitating the conversion of discontinuities left in the newly synthesised strand during replication to continuous chromosomal DNA. In particular, PRR has a critical role in the replication of damaged DNA since single-strand gaps are frequently formed opposite the damaged DNA. PRR is not necessarily S-phase-specific since the gap filling process can operate even in the G2-phase of the cell cycle, but completion of the cell cycle without complete PRR may lead to genome instability and cancer (30). In spite of the active interest and the significant investigative efforts, relatively little is known about PRR in human cells, and its main players remain to be elucidated. Since the discovery of the fork reversal activity of Rad5/HLTF, other SWI/SNF enzymes such as SMARCAL1, ZRANB3, and Rad54 have been described to exhibit similar activities, but the number of enzymes that are able to carry out fork reversal has remained limited despite the SWI/SNF protein family having numerous members (31–33). The *E. coli* RecG, yeast Mph1, human FANCM helicase, and certain RecQ helicases such as WRN and BLM can also carry out fork reversal (11,34). However, there is a main mechanistic difference between the dsDNA translocases such as the SWI/SNF enzymes and the canonical helicases in the mechanism of fork reversal since the SWI/SNF helicase-like proteins do not generate ssDNA intermediates. Instead, their dsDNA translocase activity drives the simultaneous unwinding and annealing of the nascent strands and the parental strands in the replication fork which ensures that the stalled replication forks are not processed to abortive structures. Despite accumulating data, the basis of the differences in fork reversal activities and the answer to what enables the fork reversal activity of a particular DNA translocase or helicase enzyme as well as the *in vivo* contribution of these activities to PRR is still unknown.

Here, we examine the *in vivo* role of HLTF in PRR and characterise the importance of its various protein domains in this process with a focus on its HIRAN domain. To provide structure/function insights, we carry out biochemical assays such as fork reversal, DNA translocase, PCNA ubiquitylation, and structure-specific DNA binding using purified wild-type and mutant HLTF proteins. Our observations provide strong support for the role of HLTF in PRR, clarify further the function of the HIRAN domain, and explain in general why only a subset of DNA translocases can carry out fork reversal.

## MATERIALS AND METHODS

### Plasmid constructs

To obtain HLTF with the HIRAN domain deleted (HLTF156–1009), the 156–589 amino acid region of HLTF was PCR amplified from pIL1867 (HLTF pENTR2B) using oligonucleotides O2515 (AAG AAT TCT GGA TTG GAG TAC) containing an EcoRI restriction site and O2522 (ACG CGT CGA CGC AAT AGA AAA GCG GTT TCA GAT CAG) containing a SalI site. The PCR fragment was cloned into pIL1867 EcoRI-SalI sites resulting in pIL2106, and subsequently the C-terminal part of HLTF was cloned from pIL1867 into pIL2106 using EcoRI-EcoRI digestion resulting in pIL2108 (HLTF HIRAN del pENTR2B). The wild-type HIRAN domain fragment was PCR amplified from pIL1867 using the oligonucleotides O2603 (TTT CCA TGG ATT CCG TTT TAT TTG GAA GTT TGA G) containing an NcoI restriction site and O2604 (TTT CTC GAG TCC ATG TTT CTT CAA CTG ATC TG) containing an XhoI site. The PCR product was cloned into pIL1163 (pENTR4) with NcoI-XhoI digestion resulting in pIL2174 (HIRAN domain pENTR4). For the HIRAN domain NN90,91AA point mutant fragment, mutagenic PCR was performed using oligonucleotides O2732 (CAA CGA GAT CCT GCT GCC CCT TAT GAT AAG) and O2733 (CTT ATC ATA AGG GGC AGC AGG ATC TCG TTG) resulting in pIL2734 (HIRAN domain 90/91 pENTR4). Yeast expression constructs were generated using the LR recombination system and the pIL1844 (pBJ842 GST-FLAG-Destination) vector resulting in plasmids pIL1520, pIL2111, pIL2177, pIL2735 expressing the GST-FLAG-HLTF, GST-FLAG-HLTF159–1009, GST-FLAG-HIRAN domains, and the mutant GST-FLAG-HIRAN NN90,91AA domain, respectively. The crystallization construct of the HIRAN domain (aa 56–175) was cloned from a cDNA template from the Mammalian Gene Collection (hltf.BC044659.MGC.AT70-E3.pCMV-SPORT6) into the pET28a-LIC vector (GenBank accession EF442785) using the In-Fusion CF Dry-Down PCR Cloning Kit (Clontech 639605).

### Protein purification

HLTF, MMS2-UBC13, RFC, UBA1, ubiquitin and monoubiquitylated PCNA were purified as described previously (35). HIRAN domains were purified similarly to HLTF using PreScission protease which cleaves between the GST and FLAG tags resulting in a FLAG-HIRAN domain, or using 20 mM reduced glutathione resulting in GST-FLAG-HIRAN.

Protein needed for X-ray structural determination was purified as follows. Bacterial pellets were obtained from 2-l LB media initially cultured at 37°C to an OD_600_ = 1.0, after which cells were induced with 1 mM IPTG for 16 h at 15°C. Then the bacterial pellets where sonicated and the cleared cell lysate in lysis buffer (50 mM HEPES, pH 7.5, 500 mM NaCl, 2.5 mM imidazole) was loaded onto a 5 ml TALON metal-affinity resin column equilibrated in wash buffer (50 mM HEPES, pH 7.5, 500 mM NaCl, 15 mM imidazole) at 4°C temperature. The column was washed with 50 ml of wash buffer and the protein was eluted with 10 ml of elution buffer (50 mM HEPES, pH 7.5, 500 mM NaCl, 250 mM imidazole). Upon addition of 1 unit of Thrombin protease, 2 mM β-mercaptoethanol, the eluant was incubated for 10 h at 4°C. The protein was further purified by gel filtration on a HighLoad Superdex 75 column equilibrated with Gel-filtration Buffer (20 mM sodium phosphate pH 6.3 and 100 mM NaCl). Fractions containing protein were pooled and concentrated using Amicon Ultra centrifugal filter with 3 kD cutoff membrane to a final concentration of 16 mg/ml. Protein yield was 7 mg/l of bacterial culture.

### HIRAN domain (hHLTF.56–175) crystallization

Crystals were grown at 18ºC in hanging-drop plates (Hampton, HR3-170) by mixing 2 μl of protein mix (*h*HLTF.56–175 (7 mg/ml) and 2 μl of crystallization buffer (0.1 M sodium acetate trihydrate pH 5.0, 40% (v/v) 2-methyl-2,4-pentanediol).

### HIRAN domain (aa 52–171) X-ray structure determination

Diffraction data was collected at beam-line 19ID at the *Advanced Photon Source* (*APS*) synchrotron at Argonne National Laboratory. All data sets were integrated and scaled using XDS (36). The structure was solved by molecular replacement using PHASER (search model 4XZF) followed by iterative model building using Coot (37) and maximum-likelihood refinement using REFMAC5. Statistics of data collection, processing, and refinement are provided in Supplementary Table S01.

### DNA substrates

We have previously described the generation of the plasmid-sized replication fork model substrate and the triple-helix substrate (24). To generate oligonucleotide-based DNA substrates, we annealed oligonucleotides in various combinations followed by purification from polyacrylamide gel. The oligonucleotide-based substrates were the following: O1358 (ss42-mer), O1058/O1118 (ds75-mer), O1054/O1118 (partial duplex), O1054/O1056/O1058/O1118 (fork) where the 5′ ^32^P-labeled oligonucleotides are underlined, and O3063 (ss75-mer), O3063/O1054 (ds75-mer), O1054/O1056/O1058/O3063 (fork), where O3063 is a 5′ fluorescein-labeled oligonucleotide with a sequence identical to O1118.

The sequences of oligonucleotides are given below:

O1358:
GAGAGAGAGAGAGAGAGAGAGAGAGAGAGAGAGAGAGAGAGA
O1058:
AGCTACCATGCCTGCCTCAAGAATTCGTAATATGCCTACACTGGAGTACCGGAGCATCGTCGTGACTGGGAAAAC
O1118:
GTTTTCCCAGTCACGACGATGCTCCGGTACTCCAGTGTAGGCATATTACGAATTCTTGAGGCAGGCATGGTAGCT
O1054:
AGCTACCATGCCTGCCTCAAGAATTCGTAA
O1056:
TTACGAATTCTTGAGGCAGGCATGGTAGCT
O3752:
AGCTACCATGCCTGCCTCAAGAATTCGTAATATGCCTACACTGGACCGTACTTCGCCTAGTAGACTGCCTTCCCG
O3730:
GTACCGGAGCATCGTCGTGACTGGGAAAAC
O3753:
CGGGAAGGCAGTCTACTAGGCGAAGTACGG

### PCNA polyubiquitylation, fork reversal, triple-helix displacement and *in vitro* assays

A standard *in vitro* ubiquitination reaction was carried out as described previously (20) for 1 h at 37°C using purified HLTF proteins at 20 nM concentration.

ATPase assay (10 μl) was performed in buffer A containing 20 mM Tris–HCl pH 7.0, 1 mM MgCl_2_, 10 mM KCl, 60 μg/ml BSA, 1 mM DTT, and 10% glycerol using 5–20 nM HLTF proteins, 1 mM [γ-32P]ATP and 1 μM dsDNA generated by annealing two oligonucleotides (O1249 ACACACACACACACACACACACACACACACACACACACACACACACACAC and O1250 GTGTGTGTGTGTGTGTGTGTGTGTGTGTGTGTGTGTGTGTGTGTGTGTGTGT). After incubation at 37°C for 30 min, 2 μl of each sample were spotted onto PEI cellulose F thin layer chromatography plate (Merck), followed by resolving the products using a solvent containing 1 M formic acid and 0.25 M LiCl. Products were detected using Typhoon Trio imager.

Fork reversal and triple-helix displacement assays were carried out as described previously (24). Briefly, reactions were carried out in buffer B containing 20 mM Tris–HCl, pH 7.0, 150 mM NaCl, 5 mM MgCl_2_, 5 mM ATP, 0.1 mg/ml bovine serum albumin, 1 mM DTT, and 10% glycerol with 0.5 nM ^32^P-labeled substrate DNA and HLTF protein in 5–20 nM concentration for 10 min at 37°C. After incubation, equal volumes of helicase stop buffer containing 20 mM EDTA, 2 mg/ml proteinase K, 1% sodium dodecyl sulfate, 10% glycerol, and 0.02% bromphenol blue were added followed by incubation for 5 min at 37°C. Samples were loaded onto 10% native polyacrylamide gels, and the products were separated using 1× Tris-borate buffer containing no EDTA. Assays with the plasmid-sized forks were carried out as described above, but restriction enzyme digestion was carried out before electrophoresis.

### Gel shift and DNA competition assays

Gel shift assays were carried out as described previously (25). For the DNA competition assay, first GST-FLAG-HIRAN was bound to the Cy5-labeled fork at a concentration where 95% of the DNA was bound by the HIRAN domain in binding buffer containing 20 mM HEPES 7.5, 150 mM NaCl, 1 mM DTT, 0.1 μg/mL BSA, 0,2% NP40, and 10% glycerol at room temperature for 15 min. Next, the protein-bound DNA substrate was divided and used immediately for the competition assay, in which increasing amounts of competitor fluorescein-labeled DNA substrates (0–1 μM) were added, followed by incubation at room temperature for 15 min. Samples in 0.5× Tris-borate buffer and 2.5% glycerol were loaded onto a 6% native polyacrylamide gel containing acrylamide and *N*,*N*-bis-acrylamide in 30:0.8 ratio and electrophoresed at 4°C in 0.5× Tris-borate buffer containing no EDTA, followed by visualization and analysis using Typhoon Trio Imager and its software.

### Cell culture assays

HCT116 human cells were grown in Dulbecco's modified Eagle's medium (Sigma) supplemented with 10% FCS (Sigma) at 37°C. Transfections were carried out using Lipofectamine 2000 transfection reagent (Invitrogen) according to the instructions of the manufacturer. To generate HLTF-specific shRNA-silenced stable cell lines, we cloned the HLTF-specific DNA sequences O2611 (GAT CCC CGG TGC TTT GGC CTA TAT CAt tca aga gaT GAT ATA GGC CAA AGC ACC TTT TTG GAA A) and O2612 (AGC TTT TCC AAA AAG GTG CTT TGG CCT ATA TCA tct ctt gaa TGA TAT AGG CCA AAG CAC CGG G) into the HindIII site of the shRNA-Neo plasmid, resulting in pIL2394. Next, the silencing plasmid was transfected into HCT116 cells, followed by selection using G-418 SULPHATE (Gibco, cat. no.: 11811064) to obtain stable cell lines.

The cellular localization of expressed FLAG-HLTF WT and HIRAN domain mutant proteins was analyzed by immunostaining using anti-Flag antibody (Sigma) diluted 1:300 and Cy3-conjugated anti-mouse antibody (Sigma, cat. no.: C2181) diluted 1:1000. Samples were mounted in phosphate-buffered saline (PBS) containing 25% glycerol and 1 mg/ml DAPI, followed by microscopy using an Olympus FV1000 confocal laser scanning microscope and a Leica confocal LSM.

### Sensitivity assay

A cell competition-based UV sensitivity assay was performed as described earlier (35,38). Briefly, after 24 h of transfection of wild-type and mutant HLTF expressing plasmids the cells were mixed with stable GFP-expressing HeLa cells at a ratio of 1:1 and UV-irradiated for 16h. After 7 days of culturing, the ratio of GFP-negative and GFP-positive cells (surviving cells) was determined by FACS (Guava Easy site System).

### Comet assay

The BrdU comet PRR assay was performed according to our previously published method (39). Briefly, cells were transfected for 5 h using 8 μl Lipofectamine^TM^2000 (Invitogen) reagents mixed with 5 μl plasmid DNA in 6-well plates, according to the manufacturer's instructions. After 24 h, asynchronously growing cells were pulse-labeled with BrdU for 20 min, washed with PBS, divided into two parts, and irradiated with UV or mock-treated. This labelling allowed us to follow the PRR process exclusively in S-phase cells. Next, cells were left to recover in a chasing medium supplemented with 4× dNTP for 6 h. Cells were collected by typsinization, pelleted in PBS, resuspended in low melting point agarose at 37°C and layered onto agarose-precoated frosted microscope slides. Proteins were removed by lysis in 2.5 M NaCl, 100 mM EDTA, 10 mM Tris [pH 10], 1%Triton X-100, and 0.5% N-lauroylsarcosine sodium salt. Alkaline electrophoresis was conducted in ice-cold electrophoresis buffer (0.3 M NaOH, 1 mM EDTA, pH 13) at 25 mA for 25 min. Slides were neutralised (0.4 M Tris-HCl, pH 7.4) and blocked in PBS containing 0.1% BSA and 0.1% Tween20 for 30 min. Immunostaining was performed by layering 30 μl of rat monoclonal anti-BrdU antibody (1:750, Ab-Direct Serotech) for 90 min followed by staining with Alexa Fluor 488-labeled goat anti-rat antibody (1:750, Molecular Probes, Inc.) for 2 h. Slides were analyzed by Zeiss Axioscope fluorescent microscopy and measured by Komet 5.0 (Kinetic Imaging Ltd, Liverpool, UK).

## RESULTS

### Structural determinants of HLTF function

**Figure 1.**
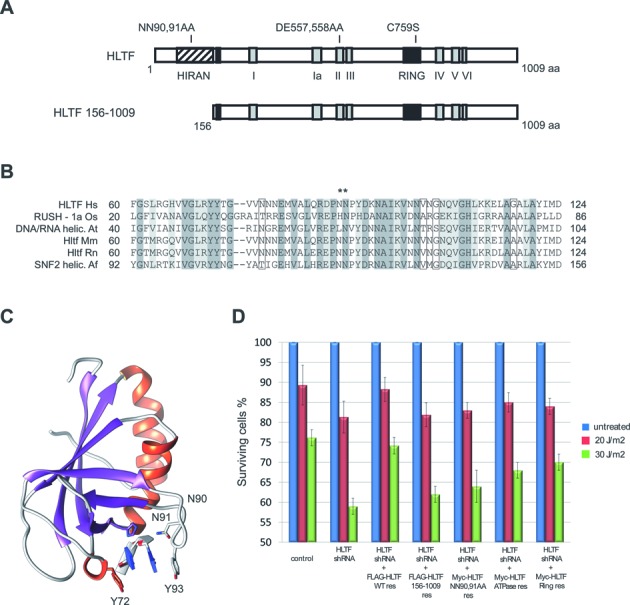
The effect of HIRAN deletion on cellular function of HLTF. (**A**) Domain structure of the HLTF protein. The SNF2-type ATPase-helicase, the C3HC4-type RING finger domain and the HIRAN motif are shown by gray boxes numbered I–VI, a black box, and a striped box, respectively. In HLTF 156–1009, the N-terminal 155 amino acids containing the whole HIRAN domain were deleted, in HLTF NN90,91AA the two asparagines in position 90 and 91 were mutated to alanines. (**B**) Multiple alignment of the HIRAN domain. Conserved residues are shaded, and asterisks indicate residues mutated to generate HLTF NN90,91AA. (**C**) The crystal structure of the HIRAN fragment (*h*HLTF.56–175) with a dinucleotide fragment reveals a six-stranded β-barrel covered with two α-helices. The DNA-binding site of the HIRAN domain is positioned in a cavity region formed by two loops and one β-strand. (**D**) Deletion or mutation of the HIRAN domain of HLTF sensitises cells to UV irradiation. Complementation of the UV sensitivity of stable shRNA-depleted HLTF knockdown HCT116 cells was tested by expressing the shRNA-resistant form of WT (HLTF WT), RING mutant (HLTF C759S), ATPase mutant (HLTF DE557,558AA), HIRAN deletion mutant (HLTF 156–1009), and HIRAN point mutant (HLTF NN90,91AA) HLTF proteins.

HLTF has three well-described domains, namely, the HIRAN domain, the SWI/SNF domain, and the C3HC4-type RING finger domain (Figure 1A). To gain more insight into the structural determinants of HLTF function, we first used computational sequence analysis and structural modeling. Computational sequence analysis suggested that the N-terminal region (∼50 residues) of HLTF preceding the HIRAN domain is mostly disordered. The linker between the HIRAN domain and the helicase region is also predicted to be largely disordered indicating that the orientation between HIRAN and the SWI/SNF2 domain might be flexible. The HLTF SWI/SNF2 region features two insertions: the RING finger domain and a long lysine/serine-rich insertion (∼320–460). This latter insertion of unknown structure features several regions of intrinsic disorder with protein-binding propensity suggesting a possible role in mediating protein-protein interactions. In contrast to the conserved RING finger domain, this sequence is missing in many homologs including both budding and fission yeast Rad5. Alignment of the HIRAN domains present in various proteins (Figure 1B) revealed its highly conserved residues, and our model indicated that the HIRAN domain is a six-stranded β-barrel covered with a single α-helix at one end and either a short helix or a loop at the other end. The HIRAN structure does not appear to be an entirely new fold since it displays some similarity with other protein structures such as Small protein B, which binds tmRNA. Furthermore, the HIRAN structure also shows a weak structural similarity to the DNA recognition domains of a couple of type II restriction enzymes such as BcnI or MvaI. Thus, structural comparisons of the HIRAN domain hint that it may bind nucleic acids.

To gain more insight into the structure of the HIRAN domain, we solved the crystal structure of the fragment *h*HLTF.56–175 (PDBID deposition 5BNH). The crystal structure of *h*HLTF.56–175 reveals a six-stranded β-barrel covered with two α-helices (Figure 1C). Interestingly, our crystal structure revealed a dinucleotide fragment that serendipitously co-crystallized with the HIRAN domain. The DNA binding site of the HIRAN domain is positioned in a cavity region formed by two loops and one β-strand. Intriguingly, this cavity, bordered by Y72 and Y93, can accommodate only one dinucleotide (single-stranded). HIRAN residues Y72 and Y93 provide further stacking interactions with the dinucleotide bases. We surmise that the distance between the tyrosines 72 and 93 acts as a ruler for this dinucleotide recognition (see Figure 1C). Given the high resolution of the crystal structure, 1.7 Å, we could identify with certainty that the dinucleotide is constituted from T and G. We suppose that the DNA fragment was co-purified with the HIRAN during the purification protocol. To ascertain the importance of this large cavity, which has the highest amino-acid conservation level, we mutated the 70, 71, 90, 91, 110, 90/110, 91/110, and 90/91 residues of HIRAN to alanine and tested the functional consequences, which revealed that only the 90/91 double mutation impaired fully the HIRAN function (see later in the text and data not shown).

### The HIRAN domain is indispensable for the cellular function of HLTF

Work in our and other laboratories revealed that the RING finger domain of HLTF is associated with ubiquitin ligase activity for PCNA polyubiquitylation, whereas the helicase domain facilitates dsDNA translocase activity; however, no function has been described for the HIRAN domain. To test the importance of the HIRAN domain in HLTF function, we generated a HIRAN deletion, HLTF156-1009, as well as a HIRAN point mutant, HLTF NN90,91AA, in addition to our previously described RING mutant, HLTF C759S, and ATPase mutant, HLTF DE557,558AA. To reveal the consequences of these mutations for the *in vivo* function of HLTF, we compared the UV sensitivity of HLTF shRNA knockdown cells expressing mutant or wild-type HLTF proteins. For this purpose, we generated stable HLTF shRNA knockdown cell lines, in which wild-type, HIRAN-mutant, ATPase mutant, or RING mutant HLTF proteins were expressed from shRNA-resistant constructs for complementation and measured their UV sensitivities (Figure 1D and Supplementary Figure S1). As expected, the HLTF-silenced cells were sensitive to UV irradiation, and wild-type HLTF was able to complement this effect. However, expression of the ATPase-mutant or the RING-mutant HLTF lowered the sensitivity of knockdown cells less than the wild-type HLTF, reflecting that the DNA translocase and ubiquitin ligase activities of HLTF are important determinants of the cellular function of HLTF, which is in line with our earlier suggestion. Importantly, the expression of HLTF lacking the HIRAN domain or point mutated in the HIRAN domain did not complement the sensitivity of the HLTF knockdown cells (Figure 1D). To rule out the possibility of the HIRAN deletion preventing HLTF nuclear localisation, we compared the localization of the wild-type, the HIRAN deletion mutant and the NN90,91AA mutant HLTF and found that all of them are localized in the nucleus (Supplementary Figure S2). In sum, our findings support the idea that the HIRAN domain of HLTF has an essential cellular role in the prevention of the deadly consequences of UV irradiation.

### The postreplication repair function of HLTF and its dependence on the HIRAN domain

**Figure 2.**
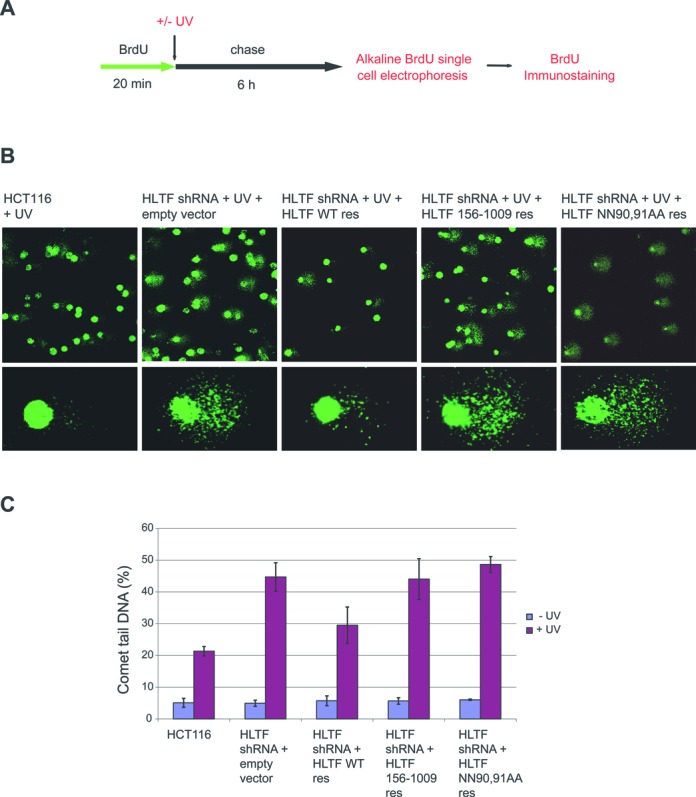
HLTF-mediated postreplication repair depends on the HIRAN domain. (**A**) Experimental setup of alkaline BrdU comet postreplication repair assay. Cells were first pulse-labeled with BrdU for 20 min, washed with PBS and UV-irradiated or mock-treated, followed by chasing by adding 4× dNTP for 6 hours before alkaline single cell electrophoresis. (**B**) Representative images of the UV-treated cells after comet assay. HLTF was stably depleted in HCT116 cells by shRNA, and for testing complementation the shRNA-resistant form of WT (HLTF WT) or HIRAN mutant HLTF 156–1009 or HLTF NN90,91AA expressing plasmids were transfected transiently. (**C**) Representation of the percentage of comet tail DNA without and after UV treatment. Standard deviations were calculated from three independent experiments.

To gain further insight into the role of HLTF and to ascertain the functional importance of its HIRAN domain, we aimed to carry out a more specific *in vivo* assay reflecting specifically the replication of damaged DNA. It is well documented that stalling of replication at unrepaired DNA lesions can result in discontinuities in the newly synthesized DNA. To overcome these discontinuities, cells have evolved mechanisms collectively referred to as postreplication repair indicating that they can also operate after the majority of DNA has been replicated and not only in the S-phase but in the G2-phases as well. To unravel the role of HLTF in postreplication repair, we used our earlier developed PRR method, which is based on the principle that if the newly replicating DNA is pulse labeled for a short period with BrdU, a thymidine analog, the maturation of the newly replicated DNA and the discontinuities left in the newly synthesized DNA can be measured using alkaline comet assay. The nascent DNA fragments can be separated from the template strand by alkaline unwinding and electrophoresis and visualized as comet tail DNA after immunostaining of the incorporated BrdU (Figure 2). If the replication process was undisturbed, after a six-hour chase period following BrdU labelling, the elongating DNA ends synthesized from neighbouring replicons abut and are sealed by DNA ligase, resulting in a continuous nascent DNA that remains in the comet head (Figure 2B and C). However, after UV irradiation, when replication fork movement is inhibited and DNA elongation is blocked, the newly synthesized DNA remains partially fragmented, indicated by the appearance of about 20 percent of the nascent strand DNA in the comet tail after a 6-hour chase period in wild-type cells (Figure 2C). Importantly, after UV irradiation, DNA maturation was more strongly inhibited in stable HLTF shRNA-silenced cells than in wild-type cells, as revealed by the increase in the percentage of comet tail DNA to 45 percent, suggesting a postreplication repair function for HLTF. This effect is attributed to the absence of the HLTF protein, since expressing wild-type shRNA-resistant HLTF in shRNA knockdown cells significantly restored DNA maturation. By contrast, the expression of the HIRAN deletion mutant or the NN90,91AA point mutant shRNA-resistant HLTF was not able to complement HLTF-silenced cells indicating that the HIRAN domain is essential for the postreplication repair function of HLTF. In contrast to damaged cells, in undamaged cells we found no difference in DNA maturation in the absence or presence of mutant or wild-type HLTF proteins, suggesting that HLTF specifically functions in the replication of damaged DNA.

### The HIRAN domain is dispensable for the ubiquitin ligase, ATPase, and dsDNA translocase enzymatic activities of HLTF

**Figure 3.**
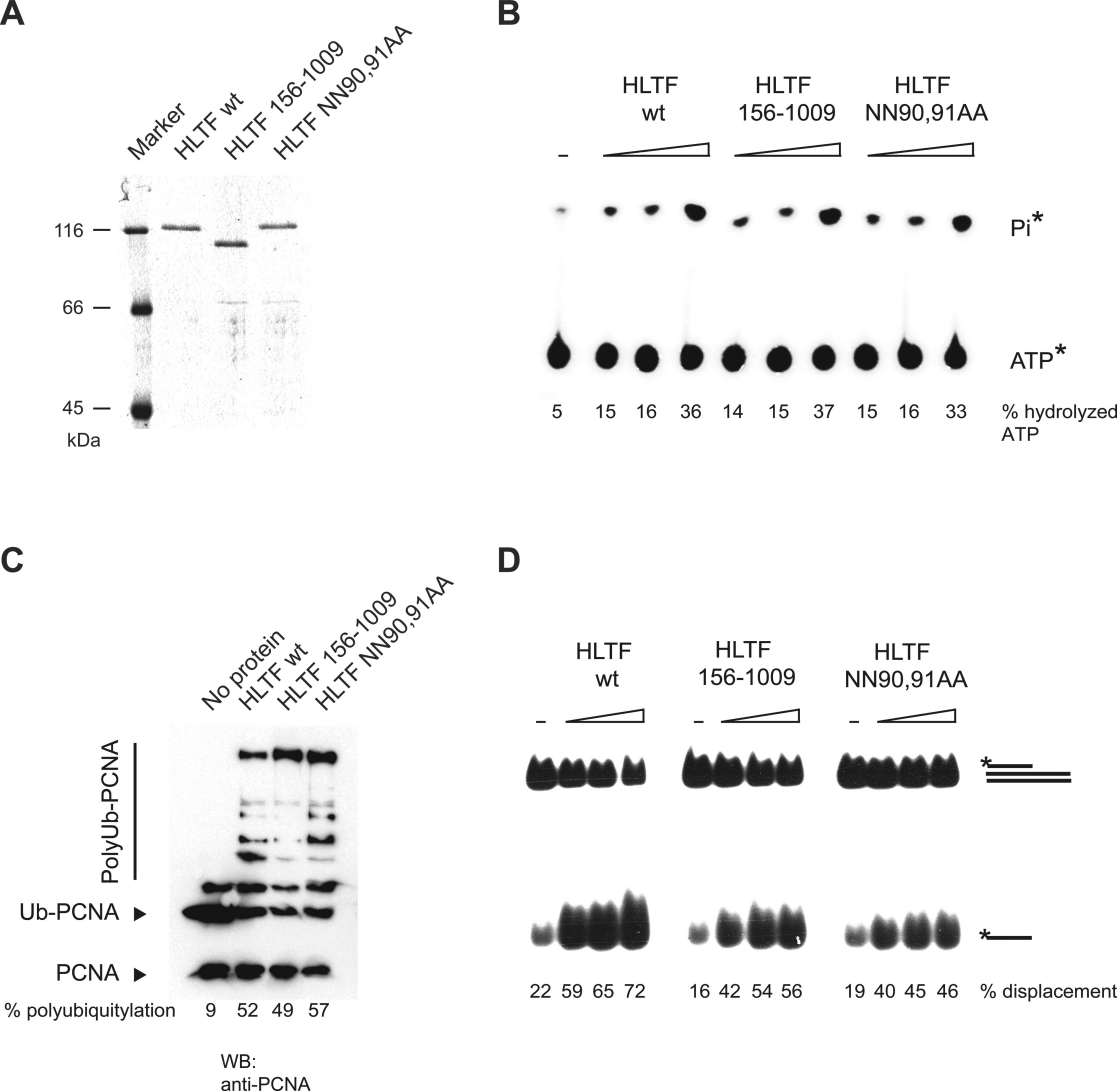
The effect of HIRAN point mutation or deletion on the enzymatic function of HLTF. (**A**) Purity of the FLAG-tagged wild-type (HLTF WT), HIRAN deletion (HLTF156–1009), and point mutant (HLTF NN90,91AA) proteins. Purified proteins were run on 8% denaturing polyacrylamide gel and stained with Coomassie blue. (**B**) Mutation of the HIRAN domain of HLTF does not compromise its ATPase activity. [γ-32P]ATP was incubated with increasing concentrations of wild-type, HIRAN domain-deleted, and HIRAN point mutant HLTF proteins in the presence of 75-mer double-stranded oligonucleotides. Hydrolyzed gamma-phosphate (Pi) was detected by thin layer chromatography. (**C**) Mutation of the HIRAN domain of HLTF does not affect its ubiquitin ligase function. PCNA was first loaded onto nicked plasmid DNA by RFC, followed by adding ubiquitin, Uba1, Rad6-Rad18, Mms2-Ubc13 and wild-type, HIRAN domain-deleted or HIRAN domain point mutant purified HLTF proteins. Ubiquitylation of PCNA was followed by western blot using anti-PCNA antibody. (**D**) Mutation of the HIRAN domain of HLTF does not affect its double-stranded DNA translocase activity. Radioactively labeled triple-helix substrate was incubated with increasing amounts of wild-type, HIRAN-deleted or HIRAN domain point mutant HLTF proteins, and the release of single-stranded DNA products was followed by native polyacrylamide gel electrophoresis.

To gain a biochemical explanation for the critical role of the HIRAN domain of HLTF in PRR, we purified the HLTF156–1009 protein completely lacking the N-terminal HIRAN domain and the point mutant HLTF NN90,91AA protein (Figure 3A). To test the effect the deletion or point mutation of HIRAN has on the basic biochemical properties of HLTF, we compared mutant and wild-type HLTF proteins in ATPase, ubiquitin ligase, and dsDNA translocase assays. We found that the HIRAN mutation did not affect the dsDNA-dependent ATPase activity of HLTF, as shown by the amount of released gamma-phosphate groups of ATP visualized by thin layer chromatography (Figure 3B). To detect the PCNA polyubiquitin ligase activity of HLTF, we loaded PCNA on circular plasmid DNA by RFC, followed by the addition of purified ubiquitin, E1, Rad6-Rad18, and Mms2-Ubc13 E2 enzymes along with wild-type or mutant HLTF and followed the reaction via Western-blot using anti-PCNA antibody. We found that the ubiquitin ligase activity of HIRAN mutant HLTF is as efficient as that of the wild-type protein (Figure 3C). For the dsDNA translocase assay, we generated a partial three-stranded DNA substrate, on whose dsDNA region HLTF can initiate translocation leading to the release of a ssDNA oligonucleotide. We established that HLTF HIRAN mutants retained a significant dsDNA translocase activity as compared to the wild-type HLTF (Figure 3D). Thus, we conclude from these experiments that the NN90,91AA point mutation or even the complete deletion of the HIRAN domain does not compromise the folding of HLTF and does not affect its ubiquitin ligase and ATPase activities while only slightly impairs its dsDNA translocase activity.

### The HIRAN domain of HLTF is necessary for replication fork regression

**Figure 4.**
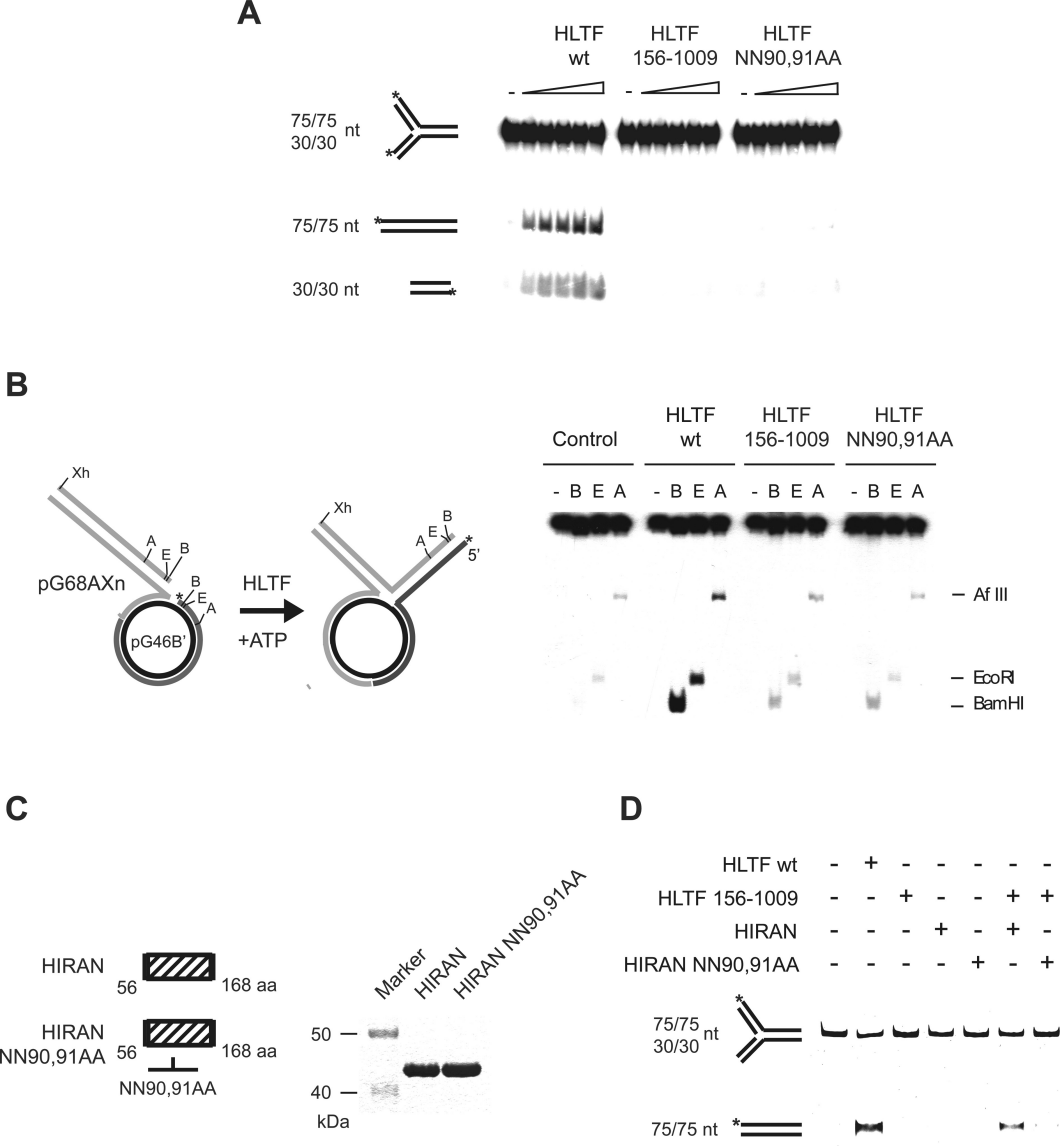
The HIRAN domain is necessary for HLTF fork regression activity. (**A**) Fork regression activity of HLTF on oligonucleotide-based replication fork-like structure. Increasing amounts of wild-type (HLTF WT), HIRAN deletion mutant (HLTF 156–1009), or HIRAN point mutant (HLTF NN9,91AA) HLTF were incubated with a radioactively labeled oligonucleotide-based replication fork-like structure. The release of the parental (75-mer) and daughter duplexes (30-mer) as products of fork regression were followed by electrophoresis on nondenaturing polyacrylamide gel. (**B**) Schematic representation of the plasmid-based model replication fork substrate used for testing HLTF fork regression activity. Fork regression activity of purified wild-type (HLTF WT), HIRAN-deleted (HLTF 156-1009), and HIRAN point mutant (NN90,91AA) HLTF proteins on radioactively labeled plasmid-based replication fork-like structure. Fork regression was revealed by digesting the products with restriction endonucleases BamHI (B), EcoRI (E) and AflIII (A). (**C**) Schematic representation and purity of wild-type and mutant HIRAN domain fragments. (**D**) Restoration of the fork regression activity of the HIRAN-deleted HLTF156-1009 protein by purified HIRAN domain fragment (containing only the 56–168 residues of HLTF). Purified wild-type or point mutant HIRAN domains were mixed with HIRAN-deleted HLTF156-1009 proteins and assayed for fork reversal activity using fluorescently labeled oligonucleotide-based replication fork-like structure. The release of parental duplex (75-mer) was monitored to reveal fork regression activity.

Having gained *in vivo* evidence for an essential role of the HIRAN domain in HLTF raised the possibility that removal of the HIRAN domain may change some replication fork-associated, specific enzymatic activities of HLTF. Our previous report indicated that HLTF can regress replication forks, for which we proposed a function in the template switching-dependent replication of damaged DNA. To test if the HIRAN domain has a role in the fork reversal activity of HLTF, we used a model replication fork substrate generated by annealing synthetic oligonucleotides. As shown in Figure 4A, fork reversal by HLTF generated two dsDNA fragments, modelling parental and daughter duplexes, which migrated faster than the fork on a non-denaturing gel. Importantly, HLTF point-mutated in its HIRAN domain (HLTF NN90,91AA) or completely lacking the HIRAN domain (HLTF156–1009) was not able to carry out fork reversal indicating a crucial role for HIRAN in this replication fork remodelling activity (Figure 4A). To rule out the possibility of an artefact due to the short size of the model fork DNA, fork regression assay was also carried out on plasmid-based fork-like structures (Figure 4B). To do so, we constructed a plasmid-sized model fork by annealing two nearly identical plasmids, resulting in a conﬁguration where the labeled ‘lagging’ strand is longer by 14 nucleotides. Conversion of this structure by fork reversal can be conveniently followed by monitoring the transfer of the radioactive label from the circular lagging arm to the linear regressed arm context by using restriction endonucleases (Figure 4B). As shown in Figure 4B, HLTF can regress the plasmid-sized fork through hundreds of bases, since at our longest checkpoint using AflIII restriction endonuclease, at a length of 863 base pairs, a regressed arm appeared readily (Figure 4B). In a parallel reaction, the HIRAN-deleted or point-mutated proteins were impaired in the reversal of the plasmid-based model replication fork (Figure 4B) revealing the critical role of the HIRAN domain. To further ascertain the crucial role of the HIRAN domain, we tested - using purified proteins - whether purified HIRAN domains can fold together with the HIRAN-deleted HLTF156-1009 protein and thus rescue its fork reversal deficiency. To this end, we expressed and purified the wild-type and the 90,91 alanine mutant HIRAN domains encompassing only amino acids 56-168 of HLTF (Figure 4C). Using an oligonucleotide-based fork, we found that mixing the wild-type but not the NN90,91AA HIRAN domains with the HIRAN-deleted HLTF resulted in reconstituted fork reversal activity (Figure 4D). In summary, our results show that the HIRAN domain is absolutely required for the fork regression activity of HLTF, and its two asparagines in positions 90 and 91 are essential for this role.

### The HIRAN domain of HLTF specifically binds to replication fork structures

**Figure 5.**
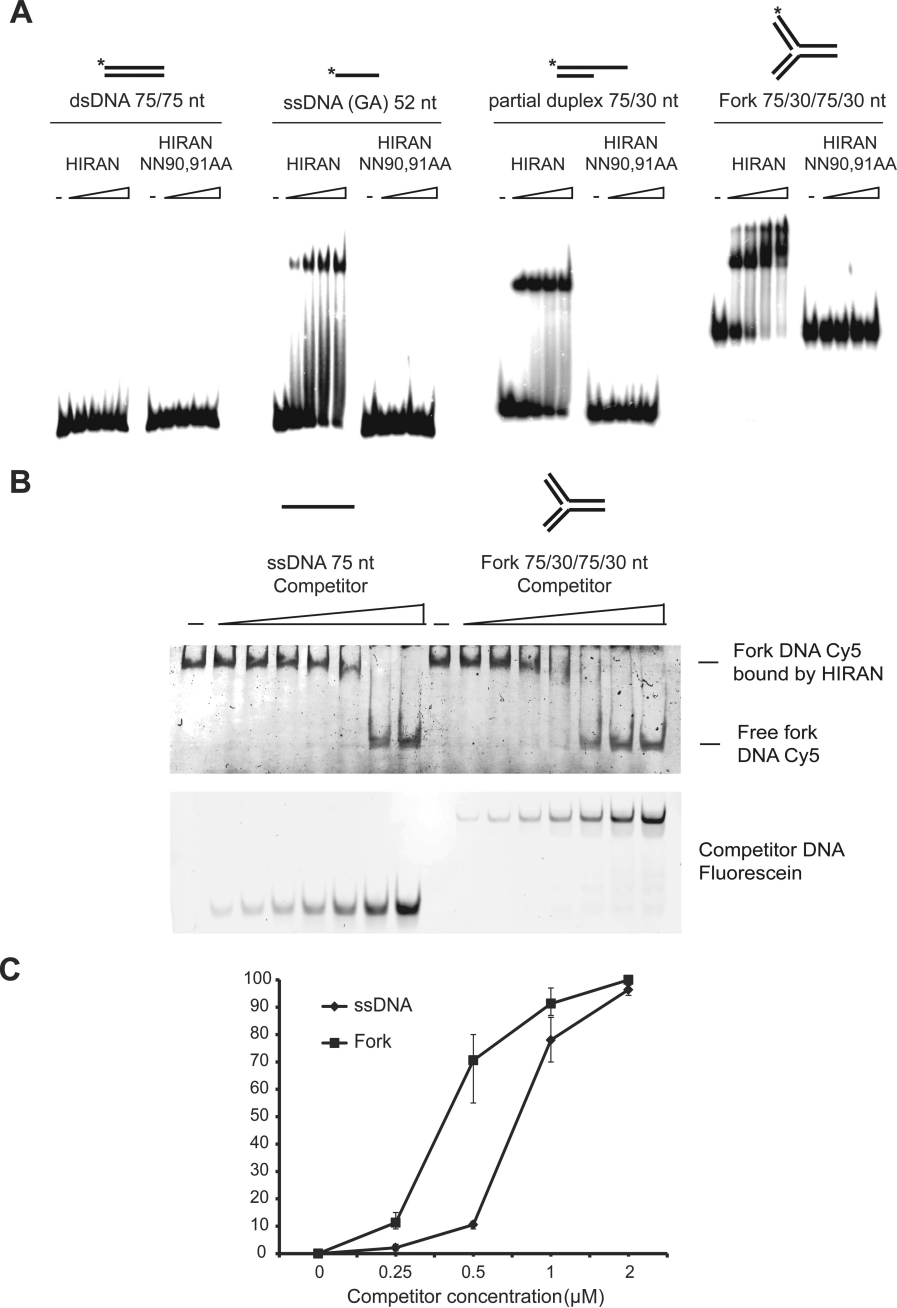
The HIRAN domain specifically binds to a replication fork-like structure. (**A**) Gel-retardation assay to reveal the binding of HLTF to various DNA substrates. Purified wild-type or mutant HIRAN domains were incubated with dsDNA, ssDNA, partial heteroduplex, and replication fork-like oligonucleotide-based DNA substrates before electrophoresis on nondenaturing acrylamide gels. (**B**) DNA competition assay. Purified HIRAN domain was preincubated with oligonucleotide-based Cy5-labeled replication fork-like substrate, followed by adding fluorescently labeled competitor ssDNA or replication fork substrates in the same increasing concentrations. After electrophoresis, the same gel was visualized first for Cy5 (upper panel) and next for fluorescent signals (lower panel). (**C**) Quantification of the DNA competition assay shown in (B).

Since deficiency in the HIRAN domain affected only fork regression but not the ubiquitin ligase or the dsDNA-dependent ATPase activities of HLTF, we hypothesized that HIRAN may lend a certain specificity to replication fork binding. To investigate this hypothesis, we tested purified HIRAN domain, both wild-type and mutant, for binding to various DNA substrates such as double-stranded, single-stranded, partial double-stranded, and replication fork-like oligonucleotide-based structures using gel retardation assay (Figure 5A). We found that the NN90,91AA mutant HIRAN domain failed to bind to any of the DNA substrates. However, the wild-type HIRAN domain exhibited a very high affinity for single-stranded DNA substrates while no affinity for dsDNA, and it also bound to a partial heteroduplex, presumably because it contained a single-stranded region (Figure 5A). Importantly, the HIRAN domain also showed a high affinity for the replication fork structure, which, however, had no single-stranded DNA region (Figure 5A). To further clarify the binding specificity, we carried out a competition assay in which a Cy5-labeled replication fork-like structure was first saturated with bound HIRAN domain then the binding was challenged by the addition of increasing amounts of fluorescein-labeled single-stranded oligonucleotides or oligonucleotide-based replication-forks (Figure 5B). We observed that the replication fork competed more efficiently for HIRAN binding than did single-stranded DNA (Figure 5B). Moreover, quantification of the competition assay also confirmed that HIRAN bound with a higher affinity to the fork than to ssDNA (Figure 5C). Thus, we conclude that although it can bind to single-stranded DNA, the HIRAN domain exhibits structure-specific binding to replication fork-like DNA structures.

## DISCUSSION

Previous studies revealed that HLTF is frequently inactivated in many tumours, indicating that loss of HLTF function promotes carcinogenesis (22,40). In concert with its potential tumour suppressor function, experiments performed by our group and others defined HLTF as a replication stress response protein that specifically acts during the replication of damaged DNA and contributes to error-free damage tolerance (19–21,24–26). We now report that HLTF plays a critical role in filling in the gaps remaining opposite the damaged DNA after the majority of DNA has been replicated, but its depletion does not slow down the overall rate of DNA maturation during the replication of undamaged DNA. We found that that the HIRAN domain of HLTF is essential for this PRR function. Significantly, we revealed that the HIRAN domain can specifically bind to the junction of a replication fork-like DNA structure, and it provides the direction for the dsDNA translocase motor domain toward the junction, which is required for HLTF-mediated fork reversal. As further support, we show that while HIRAN-deleted HLTF has dsDNA translocase activity it does not exhibit fork reversal activity and that adding the purified HIRAN fragment can complement the fork reversal activity of HIRAN-deleted HLTF. We also solved the X-ray structure of HIRAN bound to a dinucleotide and present a molecular model of how HLTF enables DNA damage bypass and replication restart.

Replication fork maintenance pathways preserve chromosome integrity; their impaired operation may generate genome rearrangements and lead to genomic disorders. Whereas in yeasts template switching is known to play an important role in DNA damage bypass - besides translesion DNA synthesis -, the degree to which template switching contributes to the above process in mammalian cells is less known, and the participants are still being identified. Although several studies confirmed the key regulatory role of the RAD6-RAD18-catalyzed ubiquitylation of the replication clamp PCNA in mammalian PRR, the effector components of the error-free PRR pathway remained unknown (2,4,7,39). To our knowledge, our present study on HLTF is the first to give direct evidence for a role of a fork reversal enzyme in mammalian PRR.

Although recent evidence confirmed that replication forks can be reversed resulting in ‘chicken foot’ structures, the physiological role of these structures is still debated (7,34). On the one hand, fork reversal was proposed to trigger nucleolytic processing of the fork particularly in the absence of normal checkpoint response. On the other hand, some authors suggested that reversal might actually protect the fork from being processed into DSB (32,41,42). Evidently, further studies are required to reveal the circumstances in which the formation of reversed forks is pathological and in which it is protective. Our data point to a protective role for HLTF-dependent fork reversal during the replication of damaged DNA; however, similar studies would be essential to test the interplay between other fork reversal enzymes such as SMARCAL1, ZRANB3, FANCM, WRN, BLOOM, and Rad54 and their contribution to PRR.

The HIRAN structure we present is basically consistent with the structures reported recently and they are in agreement regarding HIRAN as a DNA-binding domain (28,29). However, it was reported that the HIRAN domain specifically recognizes the 3′-end in a partial heteroduplex DNA and can even bind to dsDNA. In contrast, our data show that HIRAN has a high affinity for ssDNA, but it cannot bind dsDNA, and, more importantly, HIRAN displays a preferential affinity for replication fork-like junctions. The explanation for this difference may lie in our more stringent assay conditions for HIRAN-DNA binding, since we used a higher ionic strength buffer, which resembled physiological conditions more closely. The ability of HIRAN to preferentially bind to replication fork-like junctions that completely lacked single-stranded regions was unexpected, since HIRAN has no affinity for dsDNA. We suggest that this specificity of HIRAN is critical for the facilitation of the correct positioning of the HLTF motor domain to the parental dsDNA region of the fork.

**Figure 6.**
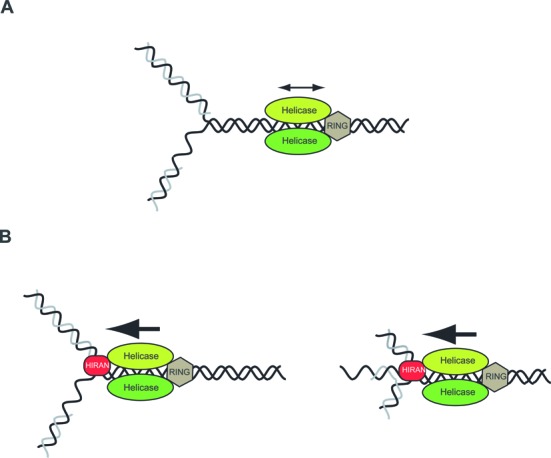
Schematic representation of the function of HIRAN. (**A**) Without the HIRAN domain, HLTF cannot be positioned at the junction point of a replication fork; although it can translocate on the dsDNA region, the junction and the two arms present a barrier for translocation into that direction. These reasons explain why HIRAN-mutated HLTF is able to displace ssDNA from a partial triple helix DNA but is unable to carry out fork reversal. (**B**) The HIRAN domain of HLTF can provide direct binding for HLTF to the junction point of the fork; serve as a pin for separating the two strands of parental/daughter dsDNA arms; orient the dsDNA translocase activity of HLTF into the right direction toward the junction. These reasons explain the critical role of HIRAN in HLTF-mediated fork reversal enabling its cellular function.

Like other SF2 helicases/translocases, HLTF is composed of two RecA-like domains that form an ATP-dependent motor enabling translocation of the protein along the dsDNA. On the other hand, the N-terminal HIRAN domain specifically determines the high affinity of HLTF for branched DNA structures and its ability to perform reversal of the replication fork. Importantly, our results indicate that HIRAN is essential only for the fork reversal activity of HLTF, but is not required for the ubiquitin ligase, DNA-dependent ATPase, and dsDNA translocase activities. From the ability of HIRAN-deleted HLTF to displace the third strand of a partial triple-stranded DNA, we conclude that HIRAN does not directly affect the motor domain of HLTF. Instead, we suggest that HIRAN provides specificity to the action of the motor domain by determining the direction for dsDNA translocation toward the junction, thereby converting the energy of ATP hydrolysis into strand separation, annealing of the daughter strands, and branch migration in the course of fork reversal. In addition to binding to the junction point and thus orienting the movement of the ATPase motor, HIRAN may also serve as a wedge at the branch point (Figure 6).

There are a number of other SF2 helicases/translocases that participate in the reversal of collapsed replication forks but none of them contain the HIRAN domain. For example, in bacterial RecG the corresponding N-terminal ‘wedge’ domain is represented by an OB-fold domain (43). Both HIRAN and OB domains are closed β-barrels, but they differ in the number of strands in a barrel: HIRAN has six while the OB-domain has five β-strands. Neither three-dimensional structures nor sequences reveal the significant similarity between the two domains. On the other hand, HIRAN lacking the N-terminal β-strand has the same topology as the canonical OB-fold including the position of the α-helix capping the β-barrel (Supplementary Figure S3). Therefore, at present it is unclear whether HIRAN is evolutionary unrelated to the OB-fold or has originated from the latter as a result of the addition of the N-terminal strand and its invasion into the β-barrel. Interestingly, HIRAN and OB domains are not the only determinants of the fork reversal function in SF2 proteins. Another domain found to be important for this function is the HARP domain. Two HARP domains are present in SMARCAL1, a DNA remodelling protein fundamental to genome integrity during replication, and a single HARP domain is found at the N-terminus of the T4 fork regression enzyme UvsW (44). The HARP domain represents a compact globular fold which consists of a five-stranded β-sheet packed against two α-helices and thus is not even remotely similar to either the OB or the HIRAN domains. The collection of ‘wedge’ domains is further extended by the α-helical domain structurally similar to the ‘thumb’ domain of DNA polymerase (45). This domain, playing a critical role in fork recognition, was first identified as an insertion between RecA-like domains in the archaeal Hef helicase. A similar domain is also present in FANCM. To further broaden the range, RecQ-like SF2 helicases BLM and WRN add to the collection yet another domain, the winged-helix (WH) domain within the RecQ family-specific region, C-terminal to the motor core (RQC) (46,47). Compiling the results regarding these specific domains of fork reversal enzymes and our finding that HIRAN-deleted HLTF loses its fork reversal activity but retains its dsDNA translocase activity indicates that, per se, the ability to translocate on dsDNA does not necessarily imply fork reversal, which is a rather specific activity requiring the specific cooperation of a motor domain and a junction-binding domain. To further support this notion, it would be of great interest to interchange the motor domains and the junction-binding domains of various fork reversal enzymes and test the resulting chimeras for their dsDNA translocase and fork reversal activities.

In summary, our data support that HLTF is an important player in PRR and its HIRAN domain is essential for this function. We suggest that when sensing a replication fork blocked by lesion, HIRAN binds to the junction of the fork and orients the motor domain into the correct position enabling HLTF to catalyse strand annealing and branch migration leading to fork reversal. In general terms, our findings explain why only a subset of DNA translocases can carry out fork reversal and highlight the importance of the presence of two DNA-binding domains in these enzymes. We conclude that in addition to the DNA-binding ATPase motor domain, fork reversal enzymes should also have a junction-specific DNA-binding domain such as the HIRAN, OB, HARP, or winged-helix (WH) domains. Based on these criteria, novel fork reversal enzymes can be identified, and future research can reveal the effect of fork reversal on DNA lesion tolerance and genome stability.

## Supplementary Material

SUPPLEMENTARY DATA

## References

[1] Ciccia A., Elledge S.J. (2010). The DNA damage response: making it safe to play with knives. Mol. Cell.

[2] Izhar L., Ziv O., Cohen I.S., Geacintov N.E., Livneh Z. (2013). Genomic assay reveals tolerance of DNA damage by both translesion DNA synthesis and homology-dependent repair in mammalian cells. Proc. Natl. Acad. Sci. U.S.A..

[3] Zhang H., Lawrence C.W. (2005). The error-free component of the RAD6/RAD18 DNA damage tolerance pathway of budding yeast employs sister-strand recombination. Proc. Natl. Acad. Sci. U.S.A..

[4] Yoon J.-H., Prakash S., Prakash L. (2012). Requirement of Rad18 protein for replication through DNA lesions in mouse and human cells. Proc. Natl. Acad. Sci. U.S.A..

[5] Prakash S., Johnson R.E., Prakash L. (2005). Eukaryotic translesion synthesis DNA polymerases: specificity of structure and function. Annu. Rev. Biochem..

[6] Silverstein T.D., Johnson R.E., Jain R., Prakash L., Prakash S., Aggarwal A.K. (2010). Structural basis for the suppression of skin cancers by DNA polymerase eta. Nature.

[7] Adar S., Izhar L., Hendel A., Geacintov N., Livneh Z. (2009). Repair of gaps opposite lesions by homologous recombination in mammalian cells. Nucleic Acids Res..

[8] Higgins N.P., Kato K., Strauss B. (1976). A model for replication repair in mammalian cells. J. Mol. Biol..

[9] Klein H.L. (2008). Molecular biology: DNA endgames. Nature.

[10] Krejci L., Van Komen S., Li Y., Villemain J., Reddy M.S., Klein H., Ellenberger T., Sung P. (2003). DNA helicase Srs2 disrupts the Rad51 presynaptic filament. Nature.

[11] Neelsen K.J., Lopes M. (2015). Replication fork reversal in eukaryotes: from dead end to dynamic response. Nat. Rev. Mol. Cell Biol..

[12] Torres-Ramos C.A., Prakash S., Prakash L. (2002). Requirement of RAD5 and MMS2 for postreplication repair of UV-damaged DNA in Saccharomyces cerevisiae. Mol. Cell. Biol..

[13] Gangavarapu V., Haracska L., Unk I., Johnson R.E., Prakash S., Prakash L. (2006). Mms2-Ubc13-dependent and -independent roles of Rad5 ubiquitin ligase in postreplication repair and translesion DNA synthesis in Saccharomyces cerevisiae. Mol. Cell. Biol..

[14] Gangavarapu V., Prakash S., Prakash L. (2007). Requirement of RAD52 group genes for postreplication repair of UV-damaged DNA in Saccharomyces cerevisiae. Mol. Cell. Biol..

[15] Blastyák A., Pintér L., Unk I., Prakash L., Prakash S., Haracska L. (2007). Yeast Rad5 protein required for postreplication repair has a DNA helicase activity specific for replication fork regression. Mol. Cell.

[16] Xue X., Choi K., Bonner J., Chiba T., Kwon Y., Xu Y., Sanchez H., Wyman C., Niu H., Zhao X. (2014). Restriction of replication fork regression activities by a conserved SMC complex. Mol. Cell.

[17] Zheng X.-F., Prakash R., Saro D., Longerich S., Niu H., Sung P. (2011). Processing of DNA structures via DNA unwinding and branch migration by the S. cerevisiae Mph1 protein. DNA Repair (Amst.).

[18] Unk I., Hajdú I., Blastyák A., Haracska L. (2010). Role of yeast Rad5 and its human orthologs, HLTF and SHPRH in DNA damage tolerance. DNA Repair (Amst.).

[19] Moldovan G.-L., D'Andrea A.D. (2011). DNA damage discrimination at stalled replication forks by the Rad5 homologs HLTF and SHPRH. Mol. Cell.

[20] Unk I., Hajdú I., Fátyol K., Hurwitz J., Yoon J.-H.J.-H., Prakash L., Prakash S., Haracska L., Hajdu I., Fatyol K. (2008). Human HLTF functions as a ubiquitin ligase for proliferating cell nuclear antigen polyubiquitination. Proc. Natl. Acad. Sci. U.S.A..

[21] Lin J.-R., Zeman M.K., Chen J.-Y., Yee M.-C., Cimprich K.A. (2011). SHPRH and HLTF act in a damage-specific manner to coordinate different forms of postreplication repair and prevent mutagenesis. Mol. Cell.

[22] Sandhu S., Wu X., Nabi Z., Rastegar M., Kung S., Mai S., Ding H. (2012). Loss of HLTF function promotes intestinal carcinogenesis. Mol. Cancer.

[23] Motegi A., Liaw H.-J., Lee K.-Y., Roest H.P., Maas A., Wu X., Moinova H., Markowitz S.D., Ding H., Hoeijmakers J.H.J. (2008). Polyubiquitination of proliferating cell nuclear antigen by HLTF and SHPRH prevents genomic instability from stalled replication forks. Proc. Natl. Acad. Sci. U.S.A..

[24] Blastyák A., Hajdú I., Unk I., Haracska L. (2010). Role of double-stranded DNA translocase activity of human HLTF in replication of damaged DNA. Mol. Cell. Biol..

[25] Achar Y.J., Balogh D., Haracska L. (2011). Coordinated protein and DNA remodeling by human HLTF on stalled replication fork. Proc. Natl. Acad. Sci. U.S.A..

[26] Burkovics P., Sebesta M., Balogh D., Haracska L., Krejci L. (2014). Strand invasion by HLTF as a mechanism for template switch in fork rescue. Nucleic Acids Res..

[27] Iyer L.M., Babu M.M., Aravind L. (2006). The HIRAN domain and recruitment of chromatin remodeling and repair activities to damaged DNA. Cell Cycle.

[28] Hishiki A., Hara K., Ikegaya Y., Yokoyama H., Shimizu T., Sato M., Hashimoto H. (2015). Structure of a Novel DNA-binding Domain of Helicase-like Transcription Factor (HLTF) and Its Functional Implication in DNA Damage Tolerance. J. Biol. Chem..

[29] Kile A.C., Chavez D.A., Bacal J., Eldirany S., Korzhnev D.M., Bezsonova I., Eichman B.F., Cimprich K.A. (2015). HLTF's ancient HIRAN domain binds 30 DNA ends to drive replication fork reversal. Mol. Cell.

[30] Karras G.I., Jentsch S. (2010). The RAD6 DNA damage tolerance pathway operates uncoupled from the replication fork and is functional beyond S phase. Cell.

[31] Ciccia A., Nimonkar A. V, Hu Y., Hajdu I., Achar Y.J., Izhar L., Petit S.A., Adamson B., Yoon J.C., Kowalczykowski S.C. (2012). Polyubiquitinated PCNA recruits the ZRANB3 translocase to maintain genomic integrity after replication stress. Mol. Cell.

[32] Bétous R., Mason A.C., Rambo R.P., Bansbach C.E., Badu-Nkansah A., Sirbu B.M., Eichman B.F., Cortez D. (2012). SMARCAL1 catalyzes fork regression and Holliday junction migration to maintain genome stability during DNA replication. Genes Dev..

[33] Shah P.P., Zheng X., Epshtein A., Carey J.N., Bishop D.K., Klein H.L. (2010). Swi2/Snf2-related translocases prevent accumulation of toxic Rad51 complexes during mitotic growth. Mol. Cell.

[34] Hu L., Kim T.M., Son M.Y., Kim S.-A., Holland C.L., Tateishi S., Kim D.H., Yew P.R., Montagna C., Dumitrache L.C. (2013). Two replication fork maintenance pathways fuse inverted repeats to rearrange chromosomes. Nature.

[35] Juhasz S., Balogh D., Hajdu I., Burkovics P., Villamil M.A., Zhuang Z., Haracska L. (2012). Characterization of human Spartan/C1orf124, an ubiquitin-PCNA interacting regulator of DNA damage tolerance. Nucleic Acids Res..

[36] Kabsch W. (2010). XDS. Acta Crystallogr. D. Biol. Crystallogr..

[37] Emsley P., Cowtan K. (2004). Coot: model-building tools for molecular graphics. Acta Crystallogr. D. Biol. Crystallogr..

[38] Smogorzewska A., Matsuoka S., Vinciguerra P., McDonald E.R., Hurov K.E., Luo J., Ballif B.A., Gygi S.P., Hofmann K., D'Andrea A.D. (2007). Identification of the FANCI protein, a monoubiquitinated FANCD2 paralog required for DNA repair. Cell.

[39] Mórocz M., Gali H., Raskó I., Downes C.S., Haracska L. (2013). Single cell analysis of human RAD18-dependent DNA post-replication repair by alkaline bromodeoxyuridine comet assay. PLoS One.

[40] Moinova H.R., Chen W.-D., Shen L., Smiraglia D., Olechnowicz J., Ravi L., Kasturi L., Myeroff L., Plass C., Parsons R. (2002). HLTF gene silencing in human colon cancer. Proc. Natl. Acad. Sci. U.S.A..

[41] Schlacher K., Christ N., Siaud N., Egashira A., Wu H., Jasin M. (2011). Double-strand break repair-independent role for BRCA2 in blocking stalled replication fork degradation by MRE11. Cell.

[42] Ray Chaudhuri A., Hashimoto Y., Herrador R., Neelsen K.J., Fachinetti D., Bermejo R., Cocito A., Costanzo V., Lopes M. (2012). Topoisomerase I poisoning results in PARP-mediated replication fork reversal. Nat. Struct. Mol. Biol..

[43] Singleton M.R., Scaife S., Wigley D.B. (2001). Structural analysis of DNA replication fork reversal by RecG. Cell.

[44] Mason A.C., Rambo R.P., Greer B., Pritchett M., Tainer J.A., Cortez D., Eichman B.F. (2014). A structure-specific nucleic acid-binding domain conserved among DNA repair proteins. Proc. Natl. Acad. Sci. U.S.A..

[45] Nishino T., Komori K., Tsuchiya D., Ishino Y., Morikawa K. (2005). Crystal structure and functional implications of Pyrococcus furiosus hef helicase domain involved in branched DNA processing. Structure.

[46] Newman J.A., Savitsky P., Allerston C.K., Bizard A.H., Özer Ö., Sarlós K., Liu Y., Pardon E., Steyaert J., Hickson I.D. (2015). Crystal structure of the Bloom's syndrome helicase indicates a role for the HRDC domain in conformational changes. Nucleic Acids Res..

[47] Swan M.K., Legris V., Tanner A., Reaper P.M., Vial S., Bordas R., Pollard J.R., Charlton P.A., Golec J.M.C., Bertrand J.A. (2014). Structure of human Bloom's syndrome helicase in complex with ADP and duplex DNA. Acta Crystallogr. D. Biol. Crystallogr..

