# Supplementary material for: Human HLTF mediates postreplication repair by its HIRAN domain-dependent replication fork remodelling
Source: Nucleic Acids Res. 2015 Sep 8;43(21):10277–91. doi: 10.1093/nar/gkv896 (PMC4666394; doi:10.1093/nar/gkv896)
Supplement: SUPPLEMENTARY DATA [file supp_43_21_10277__index.html]

Human HLTF mediates postreplication repair by its HIRAN domain-dependent replication fork remodelling — SUPPLEMENTARY DATA 

# Human HLTF mediates postreplication repair by its HIRAN domain-dependent replication fork remodelling

## SUPPLEMENTARY DATA

- SUPPLEMENTARY DATA
