# Supplementary material for: Human HLTF mediates postreplication repair by its HIRAN domain-dependent replication fork remodelling
Source: Nucleic Acids Res. 2015 Sep 8;43(21):10277–91. doi: 10.1093/nar/gkv896 (PMC4666394; doi:10.1093/nar/gkv896)
Supplement: SUPPLEMENTARY DATA [file supp_gkv896_nar-01859-d-2015-File008.pdf]

SUPPLEMENTARY DATA

Supplementary Figures

Supplementary Figure 1. The efficiency of HLTF shRNA knockdown and the expression of shRNA-resistant HLTF proteins. (A) The silencing efficiency of the HLTF shRNA construct was analysed using anti-HLTF antibody. (B) The expression of shRNA-resistant FLAG-tagged wild-type (HLTF WT) and HIRAN deletion mutant (HLTF 156-1009) and Myc-tagged wild-type, Ring mutant (HLTFC759S), ATPase mutant (HLTF DE557,558AA), and HIRAN domain point mutant (HLTF NN90,91AA) HLTF proteins was confirmed by anti-FLAG and anti-Myc antibodies, respectively. The expression levels of the different Flag- and Myc-tagged mutants are compared to those of their respective wild-type constructs. Anti-tubulin signal was used as a loading control. (C) Survival analysis of the HLTF WT constructs used in this study. FLAG- and Myc-tagged HLTF WT constructs complement HLTF knockdown at the same level.

Supplementary Figure 2. HLTF as well as HIRAN-deleted HLTF proteins exhibit nuclear localisation. Plasmids expressing wild-type (HLTF WT), HIRAN domain-deleted (HLTF 156-1009) or HIRAN domain point mutant (HLTF NN90,91AA) HLTF proteins were transiently transfected into HCT116 human cells, followed by immunostaining to reveal the localisation of HLTF and also DAPI-stained to locate the nucleus.

Supplementary Figure 3. Similarities in DNA binding by the OB domain of RecG (A) and by HIRAN (B). Aromatic residues stacked with the bases of different DNA strands are colored green.

Supplementary Table

Table S01. Data collection, phasing and refinement statistics for HLTF.56-175.

A

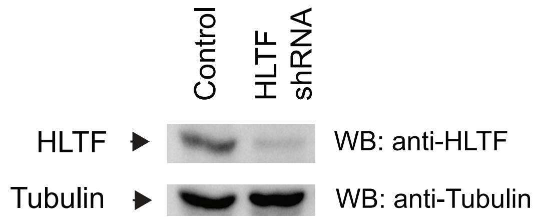

B

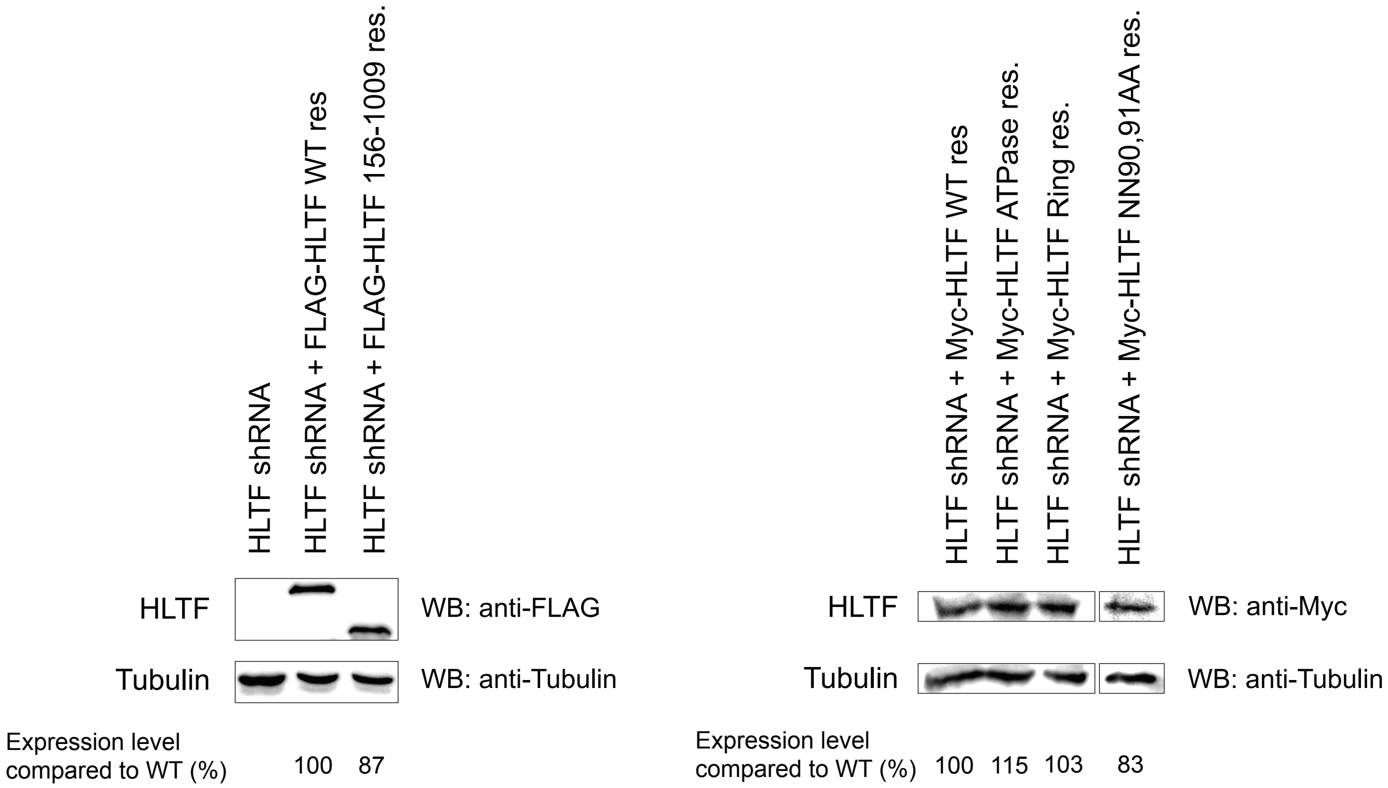

C

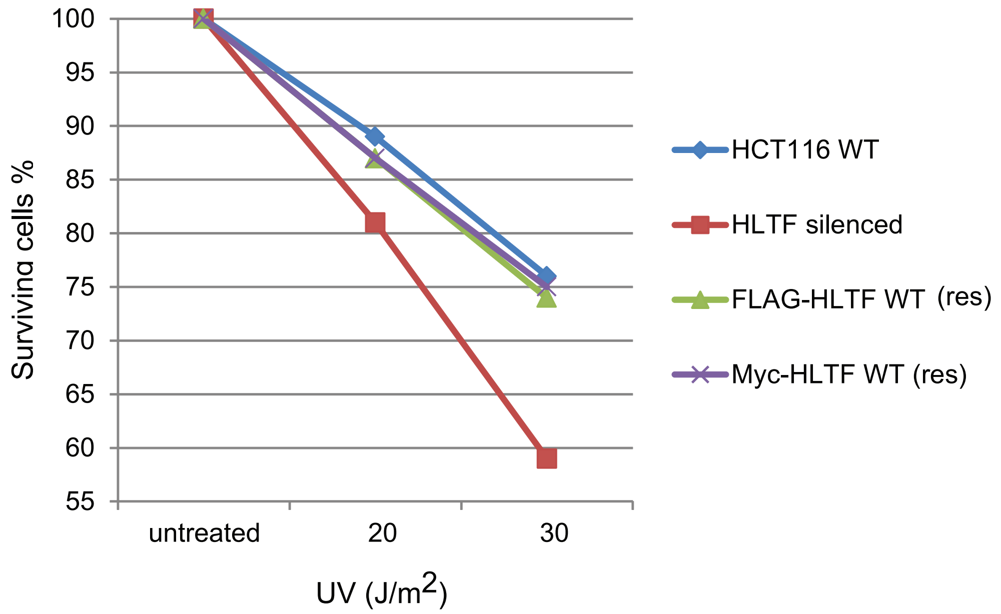

Supplementary Figure 1

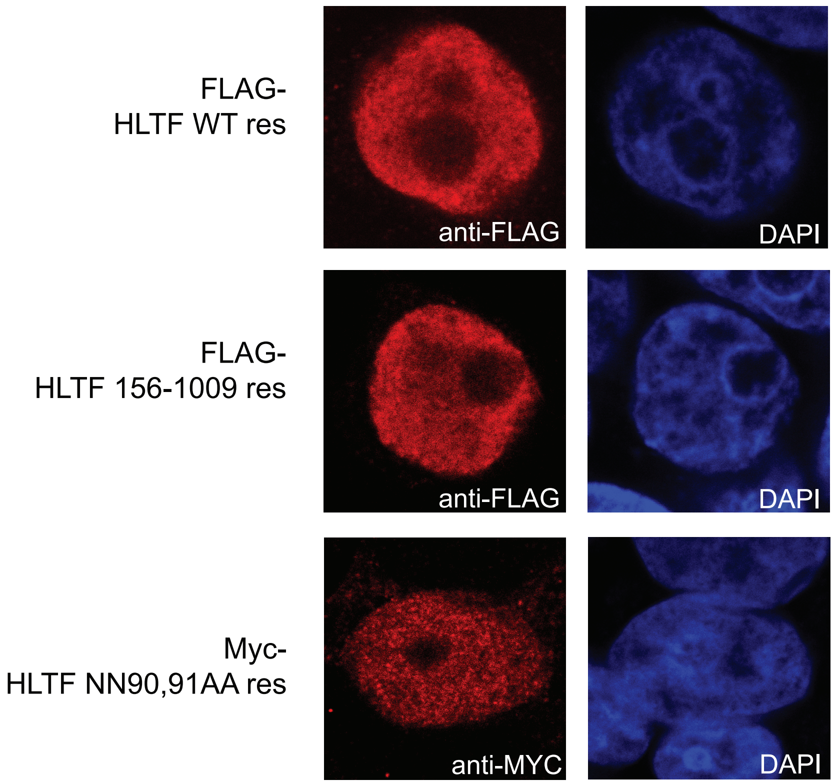

Supplementary Figure 2

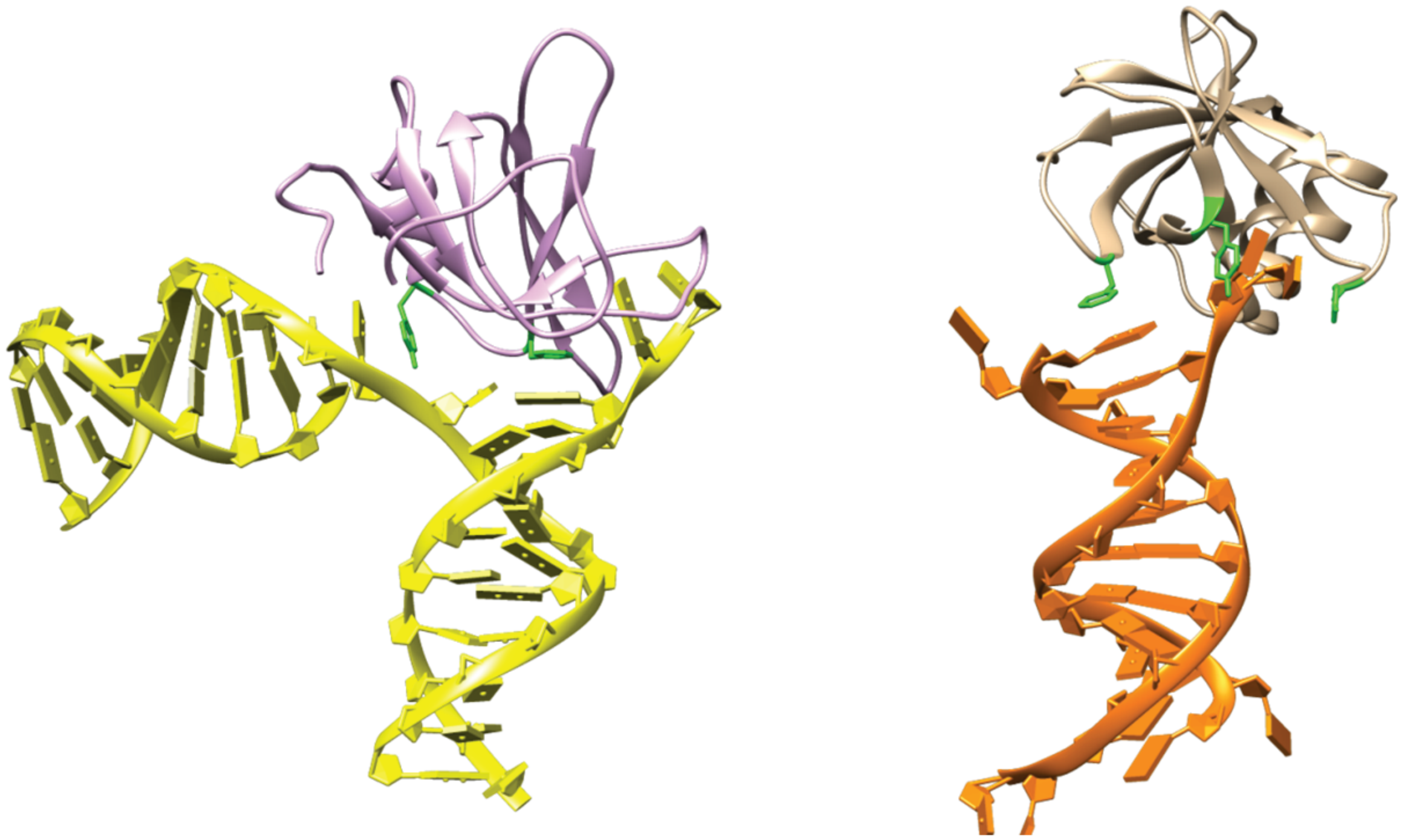

Supplementary Figure 3

	Native
**Data collection**	
Space group	$P2_12_12_1$
Cell dimensions	
a, *b*, *c* (Å)	48.47 50.12 102.76
α, β, γ (°)	90.00 90.00 90.00
Wavelength	0.979150
Resolution (Å)	51.38-1.7 (1.73-1.7)
R_{sym} or R_{merge}	0.075 (0.677)
$I / \sigma I$	9.6 (3.46)
Completeness (%)	99.6(99.5)
Redundancy	9.4(7.09)
**Refinement**	
Resolution (Å)	51.38-1.7
No. reflections	26849 (1414)
R_{work} / R_{free}	0.16655/0.21324
No. atoms	
Protein	1883
Ligand/ion	261
B-factors	22.0
R.m.s deviations	
Bond lengths (Å)	0.97
Bond angles (°)	1.06

*Values in parentheses are for highest-resolution shell.

Table S01
